# Collective behaviour across animal species

**DOI:** 10.1038/srep03723

**Published:** 2014-01-16

**Authors:** Pietro DeLellis, Giovanni Polverino, Gozde Ustuner, Nicole Abaid, Simone Macrì, Erik M. Bollt, Maurizio Porfiri

**Affiliations:** 1 Department of Electrical Engineering and Information Technology, University of Naples Federico II, Naples 80125, Italy; 2 Department of Mechanical and Aerospace Engineering, Polytechnic School of Engineering, New York University, Brooklyn, New York 11201, USA; 3 Department of Engineering Science and Mechanics, Virginia Polytechnic Institute and State University, Blacksburg, Virginia 24061, USA; 4 Section of Behavioural Neuroscience, Department of Cell Biology and Neuroscience, Istituto Superiore di Sanità, Roma 00161, Italy; 5 Department of Mathematics, Clarkson University, Potsdam, New York 13699, USA

## Abstract

We posit a new geometric perspective to define, detect, and classify inherent patterns of collective behaviour across a variety of animal species. We show that machine learning techniques, and specifically the isometric mapping algorithm, allow the identification and interpretation of different types of collective behaviour in five social animal species. These results offer a first glimpse at the transformative potential of machine learning for ethology, similar to its impact on robotics, where it enabled robots to recognize objects and navigate the environment.

Across almost every phylum within the animal kingdom, species exhibit collective behaviour at certain stages of their life cycles^1^. Collective behaviour of animal groups depends on local interactions among individuals, which drive the emergence of coordination at the group scale, for example in fish schools^2^,^3^, birds flocks^4^, insect swarms^5^, and human crowds^6^. While social interactions in select species are well studied^7^, the general mechanisms underlying collective behaviour are not fully understood^8^, partly due to the need for independently tracking large groups performing complex manoeuvres to ultimately assess species-specific patterns of group coordination^9^,^10^. Here, we establish an objective and effective method to study patterns of collective behaviour in animal groups by leveraging the evidence that we, humans, can identify and classify such patterns across animal species and without tracking every individual. To this aim, we define collective behaviour as *the manifestation of a low-dimensional manifold on which coordinated group states may be embedded*; we demonstrate a machine learning framework that identifies such low-dimensional structures to differentiate data from five social animal species. Supported by this computational framework, we expect enabling the quantitative assessment of collective behaviour through an analysis that is markedly less expensive than current methodologies, in terms of both computer and human time.

Recently, a variety of machine learning algorithms, such as support vector machines^11^, local linear embedding^12^, and principal component analysis^13^, have been developed to extract patterns from high-dimensional data sets^14^. These algorithms have been applied in a wide spectrum of science and engineering problems, including individual human recognition through biometric data^15^ and identification of trends in climate and weather^16^. Among these methods, a selection works by embedding a data set on a manifold and studying its structure as a proxy for the more complex data set. The isometric mapping algorithm (ISOMAP), originally developed for machine vision^17^, is a unique example of these methods since it preserves geodesic distances in the raw data set and in the lower-dimensional manifold coordinates it extracts, mirroring the recognition process that a human observer would experience when observing collective behaviour. Without the need of user supervision, ISOMAP generates the coordinates of the data points on the embedding manifold and a vector of residual variances, representing the proportion of data points not lying on such manifold, which is used to extract its dimensionality (see Methods).

ISOMAP has been used in refs. 18, 19 to demonstrate that collective behaviour in a system of particles is evidenced by the presence of low-dimensional embedding manifolds for the full data set. In ref. 18, low-dimensional structures are used to create a quantitative definition for collective behaviour in a simulated swarm; consistently, they are absent when collective behaviour is not observed. In ref. 19, fragmentation of a particle swarm is studied by analysing the topological features of these low-dimensional manifolds. The positive results of these analyses on simulation data serve as the basis for the animal experiments considered in this work.

Here, ISOMAP is directly applied to raw video data from five different animal taxa (ants, fish, frogs, chickens, humans) to quantitatively explore their collective behaviour in three experimental conditions (natural motion and the presence of one or two attractive stimuli) (see Methods). Using appropriately scaled physical and temporal parameters, we compare the dimensionality of the embedding manifolds identified in each data set using ISOMAP (see Methods). The experiments have the threefold aim of: i) demonstrating a proof-of-concept for the application of ISOMAP to raw video data for the study of animal behaviour, ii) seeking to understand differences in the collective behaviour across a wide evolutionary range of species through this analysis, and iii) investigating how the presence of exogenous factors, such as one or more attractive stimuli, changes the species-specific collective behaviours.

The gold standard of pattern recognition is ultimately the human brain^20^, hence we implement ISOMAP on data validated by human observers. For each species, we record videos for ten consecutive days in each of the three conditions, see the sample frames in Fig. 1. Then, each video is shown to two of the thirty observers, who are asked to ascribe a so-called collective behaviour measure (CBM), which is scaled to be comparable with the dimension of the embedding manifold, with low values indicating high interaction among individuals (see Methods). For each group of ten trials representing each species in each experimental condition, the most reliable three trials, which are those with the minimum variation between the two human observations, are selected to compare with ISOMAP. We comment that this sample size is large enough to observe differences between species, as assessed by a power analysis. The trends in the ISOMAP dimensionalities are found to be in agreement with human observations through the computation of a correlation coefficient between the results of the two analyses with each condition and species considered separately (correlation coefficient with t-test, *R*^2^ = 0.11, *d.f.* = 44, *p* < 0.05).

## Results

### ISOMAP is able to differentiate among species

Amalgamating all the selected trials from each species independently of the experimental condition, we find that the dimensionality of the embedding manifold is significantly different across species (two-way ANOVA, *F*_(4,14)_ = 4.8, *p* < 0.05, see the Statistics section for further details), see Fig. 2. Moreover, the ISOMAP dimensionality for both ants and frogs differs from all other species, representing the minimum and maximum observed values, respectively. This is consistent with the nature of social interactions in underwater frogs, which exhibit collective behaviour that is recurrent in a time window of few minutes only during their larval stage^21^ or, seasonally, during their sexual interactions^22^. In our experiments, we consider adult subjects not sexually interacting. While other forms of collective behaviour, such as collective breathing^23^, could be displayed by these subjects, the longer time scale of these phenomena would not produce appreciable variation of ISOMAP dimensionality. Indeed, the algorithm requires collective phenomena to occur several times during the video feed for them to generate low-dimensional manifolds^18^,^19^. With respect to collective breathing, we also note that the overhead view of the frogs motion is likely to minimize such sporadic phenomenon. These results indicate that this data treatment is capable of extracting differences between species' collective behaviours in the presence of variable attractive stimuli, such as food sources or the metro station entrance with respect to the humans. This analysis represents a first demonstration that a machine learning algorithm can be used to measure and characterize collective behaviour directly from raw data sets, such as video, image, or sound data, without the need of complex individual tracking. The success of ISOMAP at differentiating between species is a proof-of-concept that machine learning may offer viable tools to the study of animal behaviour.

### ISOMAP offers a biological insight into the behaviour of the selected species

**Figure 1:**
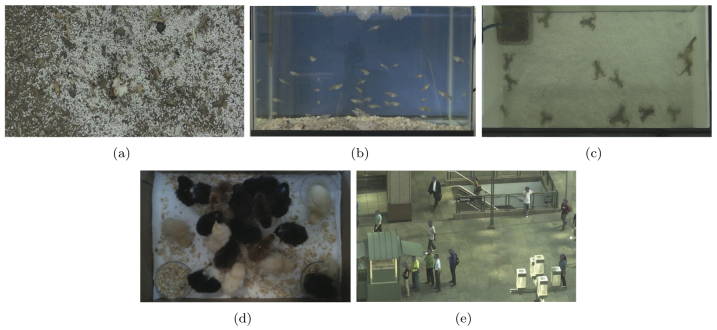
Snapshots of video data (collected by G. Ustuner) from experiments with (a) ants, (b) fish, (c) frogs, (d) chickens, and (e) humans. Human faces have been obscured to protect privacy.

**Figure 2:**
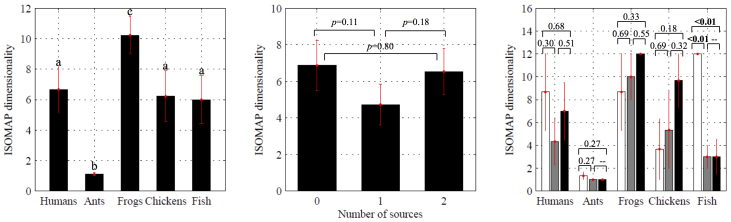
Mean ISOMAP dimensionality with all three experimental conditions combined (left), with all five species combined (middle), and for each of the five species independently (right). White, grey, and black bars represent zero, one, and two attractive stimuli, respectively. The attractive stimuli were realized as food for all species except humans, for which the attractive stimuli were the breakfast kiosk and the metro station entrance. Error bars show one standard error. Significance from post-hoc tests are indicated (Fisher's PLSD), with significant differences in bold. In the left figure, means not sharing a common superscript are significantly different in post-hoc tests.

ISOMAP is able to capture common alignment among individuals' motions, as opposed to position, since it compares images at different time intervals. As a result, ISOMAP's finding that the most coordinated animals are the ants supports the biological observations that ants exhibit highly aligned motion following pheromone trails^24^. The behaviour of ants compared to humans further demonstrates that ISOMAP identifies aligned motion. Video data on ants and humans appear superficially similar since individuals in both species tend to follow in the paths of their peers. While ants have few physical obstacles and generally follow straight paths through the video frame, the humans' domain includes many obstacles, such as the metro station entrance, coffee kiosk, and various posts and poles affixed to the ground which must be circumnavigated, see Fig. 1. Thus, ISOMAP finds that ants have organized behaviour due to their highly linear motion similarly to the results on numerical simulations in ref. 18. While the behaviour of humans is generally along one-dimensional trajectories, individuals are seldom aligned in the frame, causing ISOMAP to assess the motion as higher dimensional.

### The presence of stimuli may influence ISOMAP dimensionality

Combining data from all the species, we find that the ISOMAP dimensionality is not significantly different across experimental conditions (two-way ANOVA, *F*_(2,14)_ = 1.6, *p* = 0.23). To delve further into the behaviour of each species as the condition is varied, we perform one-way ANOVAs with condition and ISOMAP dimensionality as the independent and dependent variables, respectively. We find that collective behaviour across the three experimental conditions varies significantly only for fish (one-way ANOVA, *F*_(2,3)_ = 40.3, *p* < 0.01). Specifically, we observe significant differences comparing zero and non-zero stimulus conditions, as the presence of the stimuli induces a schooling behaviour that is not naturally present in the group.

## Discussion

In summary, the results of the ISOMAP analysis offer a first application of machine learning techniques to interpret different types of collective behaviour in five social animal species. We find that species, and sometimes, experimental conditions, are differentiated by the algorithm independently of user supervision. The findings of ISOMAP, which draws on aligned motion to identify collective behaviour in this work, are explained in terms of the species selected for this study. Furthermore, the effectiveness of the analysis is confirmed through a comparison with human observations.

One major question that arises from these results, and their comparison to human measurements of CBM, is what features of video data is ISOMAP capturing as collective behaviour to generate low-dimensional embedding manifolds. In principle, ISOMAP may identify low-dimensional embedding manifolds even in the absence of interactions, for example if the motion of individuals were restricted to a few pathways. To remedy this potential confound, the experiments are designed to mandate a sufficiently dense population of individuals occupying the recording space, thus ensuring some degree of interaction in the group. In particular, the sides of each experimental domain are never larger than 35 body lengths, so that each species can perceive the whole domain while manifesting some interaction to avoid collisions with conspecifics. In addition, the trends we observe in ISOMAP dimensionalities are in agreement with the literature on species-specific social behaviour, thus supporting the use of the proposed empirical definition of collective behaviour. For instance, the species known to exhibit asocial behaviour within the select observation time (underwater frogs) shows significantly larger ISOMAP dimensionality than the other species considered in this study. While we cannot always dismiss the possibility that boundary effects could have contributed to the observed variations, we expect that both the experimental design and the ISOMAP algorithm mitigated their role. Even if ants and humans were observed in an unenclosed environment, differently than fish, frogs, and chickens that were restricted to a fixed area, the size of the groups were balanced in time through the continuous influx of individuals through the metro station entrance and the anthill. In this context, the implementation of the ISOMAP algorithm on simulation data of groups of self-propelled particles of a fixed numerosity shows that the relationship between the dimensionality of the embedding manifold and group coordination does not vary if reflective (particles bounce back from the walls of a restricted area) or periodic (particles that leave the unenclosed environment from a wall reappear on the opposite wall) boundary conditions are used in the analysis^18^,^19^.

While we expect that the proposed approach can be adapted for the analysis of a broad spectrum of collective behaviours, such as coordinated chirping in crickets^25^ and synchronous flashing in firefly groups^26^, this study is focused on collective behaviours that are manifested in the form of aligned motion and aggregated positions. This restriction is implicit in the training of human observers, who watch a variety of particle swarms with varying alignment and grouping, and in the type of data, species, and behaviours we target. In future work, we will seek to extend the approach to other instances of collective behaviour, and, at the same time, we will explore alternative dimensionality-reduction or pattern recognition algorithms to highlight other salient mechanism of group coordination. In addition, we will leverage ISOMAP ability to preserve geodesic distances to understand the relation between topological features of the embedding manifolds and tangible modes of collective behaviour. The results of this work are expected to bridge the gap between manual and automated data analysis, which will ultimately contribute to the systematic definition of collective behaviour across diverse animal groups.

## Methods

### Animals and apparatuses

Mosquitofish (*Gambusia affinis*) and juvenile chickens (*Gallus domesticus*) were purchased from online aquarium and poultry sources (LiveAquaria.com, Rhinelander, WI, 54501 USA and Meyer Hatchery, 626 Ohio, 89 Polk, OH, 44866 USA), respectively. Aquatic frogs (*Xenopus laevis*) were purchased from a local aquarium store (Petland Discounts, Brooklyn, NY, 11201 USA). Humans (*Homo sapiens*) were observed in a public space near the Polytechnic School of Engineering, New York University (NYU-Poly) at Six MetroTech Center (Brooklyn, NY, 11201 USA). Ants (*Tetramorium caespitum*) were observed in a Brooklyn public park (Ave K and Ocean Pkwy, Brooklyn, NY, 11230 USA).

Populations of fish, frogs, and chickens were observed in captive conditions. Fish and frogs were housed in a vivarium in the Department of Mechanical and Aerospace Engineering at NYU-Poly. Chickens were maintained in a private facility near NYU-Poly. Approximately 20 individuals of each species were procured and subsequently acclimated for a minimum of 12 days prior to the experimental campaign. Fish and frog populations were each housed in holding tanks 50 cm long, 25 cm wide, and 30 cm high and with 36 litre volume. Chickens were kept in a cubic structure with side length 43 cm and open top face. For all species, water and air temperature were maintained constant at 26 ± 2°C and illumination was provided by diffused lights for ten hours each day in accordance with the circadian rhythm of these species. Animals were fed daily during or after the conclusion of the daily experimental session. Fish were fed with commercial flake food (Hagen Corp., Nutrafin max, USA), frogs with frozen bloodworms, and chickens with commercial granulated food (Chick Starter-200lbs, P/U, Meyer Hatchery, USA).

The housing structures of captive fish, frog, and chicken populations served as experimental apparatuses. A digital video-camera (Canon, Vixia HG20, Japan) was suspended above test structure for data acquisition of frog and chicken behaviour. For fish experiments, a lateral view of the aquarium was recorded to capture the primary motion of mosquitofish toward the food sources (along the horizontal axis of the tank and vertically toward the water surface) and minimize the effect of the produced surface wavelets on ISOMAP analysis. In each case, the camera was placed sufficiently far from the experimental domain to capture the entire space accessible by the animals. The attractive stimuli for fish were flake food placed in plastic rings, which floated on the water surface and were adhered to the aquarium side. For frogs, the attractive stimuli were bloodworms placed on the aquarium floor. Since the bloodworms were negatively buoyant, they did not require any physical constraint. The attractive stimuli used for chickens were commercial feed placed in standard feed bowls.

Humans and ants were observed in uncontrolled conditions and their behaviour was recorded by the camera suspended to provide a bird's eye view of the experimental area. The height of the camera was sufficient to capture individuals near the two attractive stimuli when present. For ants, small pieces of food were placed close to an anthill as attractive stimuli. For humans, the attractive stimuli were a breakfast kiosk and a metro station entrance present in the physical landscape. Sample experimental video frames for each species are shown in Fig. 1.

### Experimental procedure

Animal species were chosen based on their availability, cost, and ease of management in captivity. In particular, laboratory experiments were favoured for fish, frogs, and chickens to optimize control of experimental conditions^27^.

Experiments were conducted according to the following procedure. The social behaviour of each animal group was analysed in three experimental conditions. The baseline condition measured animal behaviour with no stimuli and the two other conditions included the presence of one and two attractive stimuli. The experiments were designed to understand differences in the collective behaviour across species and investigate how the presence of one or more attractive stimuli changes the natural collective behaviour in each species. The attractive stimuli were realized as food for all species except humans, for which the attractive stimuli were the breakfast kiosk and the metro station entrance. These locations are active hubs during the time when the data was acquired, see Fig. 1(e). The experimental setup was adapted with respect to the average body dimension and maximum speed of each species. That is, the parameters for the recording time, sampling rate, and visual field size were calculated before the start of the experiments to standardize the recordings. Also standardized was the distance between the two attractive stimuli when present. In this case, the reference distance was based on the distance observed between the kiosk and the metro station (7 m) used as attractive stimuli in the experiment with humans. The ratio between this distance and the mean body length was kept constant across species. Table 1 reports the experimental parameters. All species were experimentally observed daily for ten consecutive days during the same time window and from the same perspective and position.

The experiment described in this work was approved by NYU-Poly Animal Welfare Oversight Committee AWOC-2012-102. Both the housing and the experimental procedure were designed to minimize stress in the animals.

### Data analysis

As in ref. 18, we define collective behaviour as the existence of a low-dimensional stable invariant manifold in the space of the trajectories of a system of particles. We used ISOMAP^17^ to detect the existence of group coordination and analysis by human observers for validation. ISOMAP was applied directly to raw video data, since low-dimensionality was expected to manifest even in the high-dimensional space of images in which collective dynamics was observed^18^. In the following, we describe the treatment of video data and the statistics used to compare results.

### Video data

Experimental videos were recorded at thirty frames per second and converted to image frames using MTS converter and Avidemux software. The data were appropriately sampled so that, in each experiment, the differences between one frame and the next one were comparable between species with respect to each species' characteristic speed. In other words, the sampling period *s* was selected to be inversely proportional to the speed *v*_pix_ of each species recorded. The reference species was taken to be fish in the presence of attractive stimuli, since they represented the fastest individuals with respect to their body length. These experiments were sampled at a period of 1 frame. Sampling periods for all other species and experimental conditions were taken accordingly and are reported in Table 1. To compensate for the slower speed of fish in the absence of stimuli, the sampling period was increased as detailed in Table 1; none of the other species displayed such a marked speed change across the three conditions. After sampling each trial video, the data set was a series of 960 × 640 pixel grey scale images whose pixel values were in the range 1 to 256.

### ISOMAP algorithm

ISOMAP seeks a low-dimensional stable invariant manifold in the space of trajectories of a system of particles. In ref. 18, the presence of such a manifold has been shown to be a footprint of collective behaviour for simulation data on self-propelled data. Here, ISOMAP was directly applied on the grey scale images extracted from the recorded videos. In this context, the motion of the individuals corresponds to a change in the pixel values between frames. The input to ISOMAP is the data set 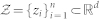, where, in our experiments, *n* = 900 was the number of frames, *d* = 614400 was the number of pixels according to the image resolution, and *z_i_* ∈ {0, 1,…, 255}. ISOMAP aims at building a corresponding data set 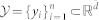, which is embedded in an invariant manifold, and assessing if 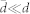.

The embedding manifold can be represented through the parametrization 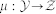, where each *j*-th coordinate of the *i*-th data point is parametrized as 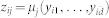, for *j* = 1,…, *d*, and for each data point *i* = 1,…, *n*. Namely, the second subscript is used to identify vector components. The main steps of the algorithm are:

1. Construction of a neighbours graph to approximate the embedding manifold. We consider the graph 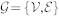, where the elements of the set of vertices 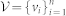 match the data points 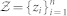, while the elements of the set of edges 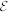 are unordered pairs of vertices in the graph. We assign edges to connect vertices that are *ν*-nearest neighbours. Specifically, we construct a *ν*-nearest neighbours graph, which consists of edges {*v_i_*, *v_j_*} corresponding to the *ν*-closest data points *z_j_* to *z_i_*, for each *i* = 1,…, *n*, with respect to the Euclidean distance in the ambient space (the pixels space), denoted by 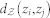. 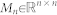 is defined as the matrix encoding the weighted graph of intrinsic manifold distances corresponding to the graph 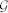, whose *ij*-th entry is *M_n_*(*i*, *j*). For each edge 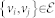, we define the distances 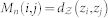 and, for all 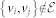, we set *M_n_*(*i*, *j*) = ∞ so as to prevent jumps between branches of the underlying manifold.
2. *Computation of the graph geodesic matrix to approximate the geodesics of the manifold*. This is performed using a well-established method to compute shortest paths, such as Floyd's algorithm^28^. From *M_n_*, we compute an approximate geodesic distance matrix 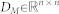, whose *ij*-th entry is the shortest weighted path length from *v_i_* to *v_j_*, being an approximation of manifold geodesic distances.
3. *Approximation of the manifold distance by ν-nearest neighbour distance*. The distance matrix *D_M_* computed in the previous step is used to approximate the geodesic distances of the manifold between *z_i_* and *z_j_* by the graph distance between *v_i_* and *v_j_*. If the data density is too low, then some neighbours might be on separate manifold branches, resulting in a poor representation of the manifold. The number of nearest neighbors was set to 11 based on a preliminary analysis on a few randomly selected videos, showing that similar embedding manifold topologies are consistently recovered for values of the number of nearest neighbors ranging from 7 to 15. A similar parameter selection was used in refs. 18, 19.
4. *Computation of the projective variables *
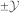
* applying the classical multidimensional scaling (MDS) method on the matrix D_M_*. Classical MDS^29^ computes a matrix of dissimilarities between pairs of items that minimizes a loss function, which here is the distance in the embedded manifold. While MDS is generally considered to be linear, the steps to produce a graph approximating the geodesic distances are decidedly nonlinear, thus making ISOMAP a nonlinear manifold learning method. For a survey of MDS, we refer the reader to ref. 18.

The outputs of ISOMAP are the transformed data points on an embedding manifold for the input data set 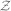 and the vector *R* of residual variances, which represents the proportion of data points not lying on such manifold. The norm of the residual variances is used to determine the dimensionality of the embedding manifold that well approximates 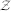. Specifically, we say that this dimensionality corresponds to the minimum value 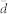 such that 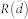 is less than 0.05.

**Figure 3:**
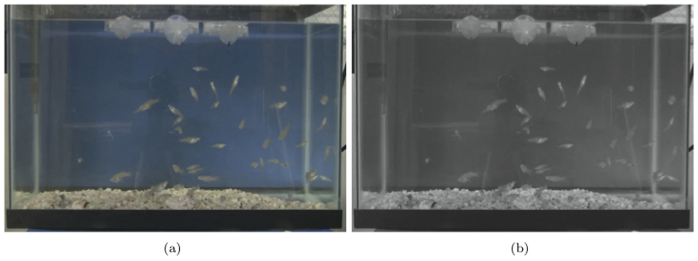
Video data for ISOMAP. Samples of (a) colour and (b) grey scale frames (taken by G. Ustuner) from a representative experiment.

**Figure 4:**
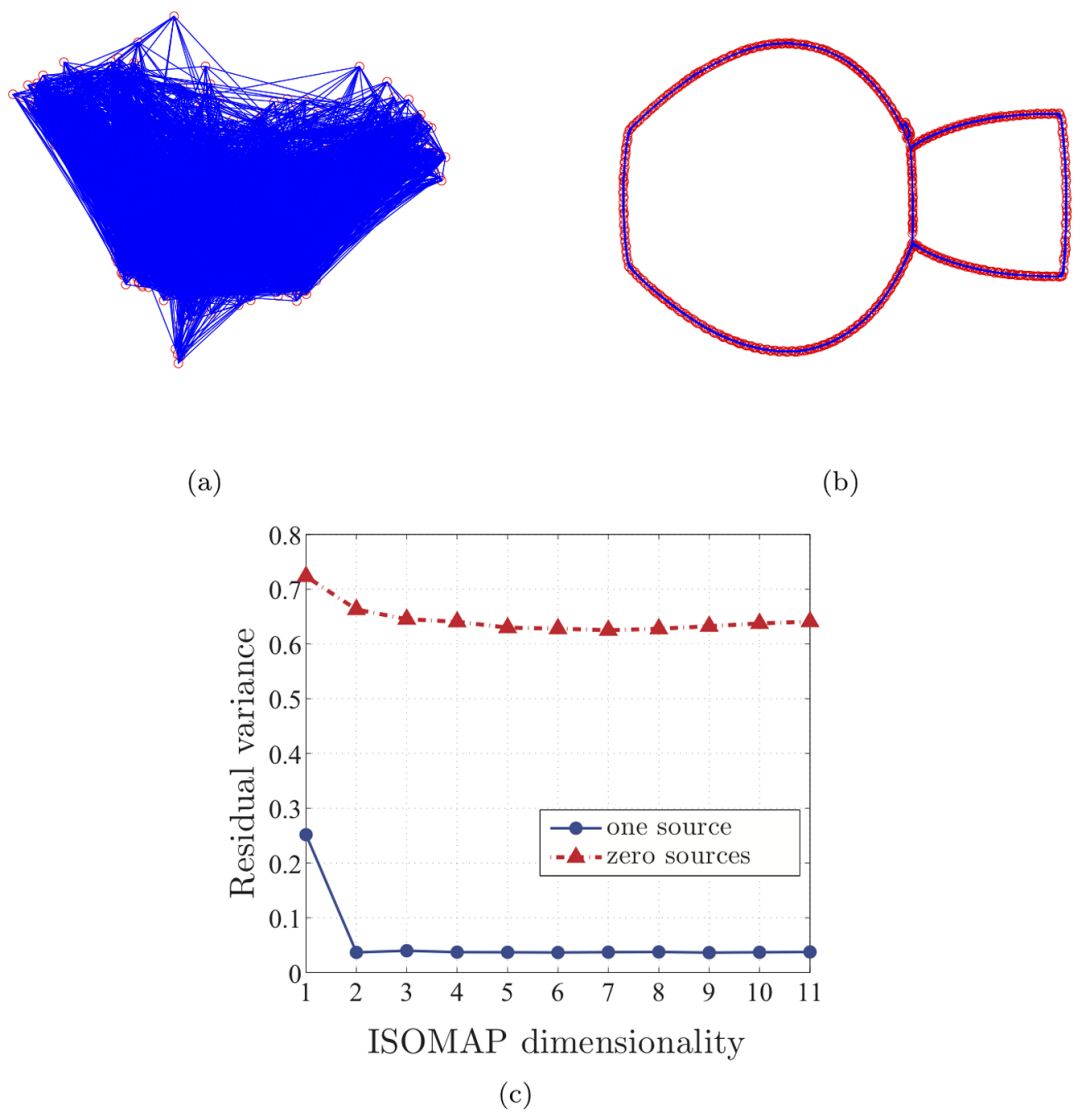
Two-dimensional embedding manifolds generated by ISOMAP for a representative trial from fish experiments with zero (a) and one (b) attractive stimuli, as well as the corresponding residual variances (c).

To provide some insight on the way ISOMAP works and on how its outputs are evaluated, we focus on two representative trials selected from the fish experiments, with zero and one attractive stimuli, respectively. Firstly, the image frames are converted to grey scale pictures, see for instance Figs. 3(a) and 3(b). Following the steps of the algorithm, an approximate geodesic distance between every pair of frames is built, where the distances are evaluated in terms of pixel values. Then, using the MDS method, the transformed 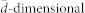 data points of the embedding manifold are obtained, with 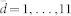. In addition, the vector 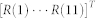 of residual variances are computed, which gives a measure of the accuracy of the manifold approximation. Namely, 0 ≤ *R*(*d*) ≤ 1, with *R*(*d*) = 1 corresponding to the worst approximation and *R*(*d*) = 0 to a perfect approximation. In Figs. 4(a)–4(c), we report the two-dimensional embedding manifolds and the residual variances for the selected trials. Fig. 4(a) shows that the dimensionality 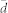 is higher than 2, while from Fig. 4(c), we observe that *R*(*d*) > 0.05 for all *d* = 1,…, 11, and therefore 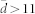. When a stimulus is added, the embedding manifold is clearly two-dimensional, as illustrated in Figs. 4(b) and 4(c), where dimension equal to 2 is detected at *R*(2) = 0.04. We comment that each animal's body geometry, including limb placement and movement, may potentially impact the residual variances found by ISOMAP. However, this effect is expected to be dominated by the bodylength-scale translations of the animals that underlie the overall group motion. This assumption is supported by ref. 18, which shows that, for a group of self-propelled particles, a small disturbance on their orientation produces a minimal increase in the residual variance, without impacting the ISOMAP dimensionality.

### Human observers

For each experimental trial, we tasked human observers with assessing the level of coordination in the animal groups. Thirty human observers participated in these experiments, which comprised a training phase and an experimental phase. During the training phase, observers were shown nine thirty seconds video clips of the particle swarm from ref. 19 moving with all nine combinations of zero, one, and two attractive stimuli and low, moderate, and high random noise. As the training videos played, the observer was provided with a collective behaviour measure (CBM) of each video, quantifying the degree of interaction and the resulting level of coordination of the group. Such measure was based on the ISOMAP dimensionality computed following the approach in refs. 18, 19, wherein it was demonstrated that such measure is a valid indicator of the group polarization, whereby low polarization relates to high CBM and high polarization results into low CBM. During the subsequent experimental phase, the observers were each shown ten experimental trials-two of the three conditions from each of the species, in colour scale rather than grey scale to offer them a more natural view of the group behaviour- and asked to ascribe a CBM to each. The CBM ranged from one to twelve, where one indicated the most interaction among individuals and twelve the least. Similarly to the ISOMAP dimensionality, low values for the CBM indicate high coordination in the system. Each trial was analysed by two independent observers. We emphasize that ISOMAP would give indistinguishable results in the case of colour or grey images. In fact, RGB images are largely redundant in terms of ISOMAP due to the strong correlation between the three channels. Hence, we provided ISOMAP with gray scale images to minimize the computational burden.

### Statistics

For each species, we obtained ten replicate trials in each of the three conditions. Each of these trials was analysed individually by two of the thirty observers to garner the CBM for each experimental conditions. Each observer analysed a total of ten videos. For each experimental condition and species, from the ten trials, we selected the three with the minimum variation between the two human observers to test with the ISOMAP code from ref. 18 using *ν* = 11 to yield an ISOMAP dimensionality. ISOMAP dimensionality and CBM were compared with a correlation coefficient *r* between the two data sets and its significance was assessed using a t-test with the t-statistic 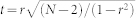. In all tests, *p* < 0.05 was taken as significant. To analyse the effect of species and experimental condition for the ISOMAP analysis, we performed a two-way analyses of variance (ANOVA)^30^ with condition and species as the independent variables and the ISOMAP dimensionality as the dependent variable. Furthermore, an additional one-way ANOVA was executed within each species to delve into the species-specific effects of condition. In these one-way ANOVAs, condition was the dependent variable and the ISOMAP dimensionality was the independent variables. For all ANOVAs, post-hoc tests were performed using Fisher's protected least squares differences (PLSD) when a significant main effect was observed.

## Author Contributions

P.D., G.P., N.A. and M.P. conceived the paper and designed the experiments. P.D., G.P., N.A., E.M.B. and M.P. wrote the main manuscript. G.U. conducted the experiments. P.D., G.P., N.A., S.M. and M.P. analysed the data. All authors reviewed the manuscript.

## Supplementary Material

Supplementary Information Sample training video 1

Supplementary Information Sample training video 2

Supplementary Information Sample training video 3

## Figures and Tables

**Table 1:** Experimental parameters: *T* is the video length in seconds, *s* is the sampling period in seconds, and *σ* is the distance between the two attractive stimuli in centimetres. In the fish experiments, *v*_pix_ is reduced by a factor of twenty for the condition in which stimuli are absent, and we set *T* = 600 and *s* = 20

	Ants	Fish	Frogs	Chickens	Humans
*T*	390	30	480	480	600
*s*	13	1	16	16	20
*σ*	1	16	16	35	700
